# Supplementary material for: MetaRibo-Seq measures translation in microbiomes
Source: Nat Commun. 2020 Jun 29;11:3268. doi: 10.1038/s41467-020-17081-z (PMC7324362; doi:10.1038/s41467-020-17081-z)
Supplement: Supplementary file 10 — Supplementary Data 7 [file 41467_2020_17081_MOESM10_ESM.zip › File2/Confidence_VeryHigh_Taxonomy/305717_out.krona.html]

Javascript must be enabled to view this page.

members
magnitude
magnitudeUnassigned
count
unassigned
taxon
rank

305717\_out

15

2
superkingdom
15

15
phylum
976

200643
class
15

15
order
171549

1
family
815

1
genus
816


SRS017307\_contig\_number\_20627
671267
species
1

13
family
171552


SRS049959\_contig\_number\_contig-100\_14221.196901
838
1
13
genus

species

SRS015794\_contig\_number\_558
2292365
1

165179

SRS011134\_contig\_number\_41021SRS013521\_contig\_number\_543SRS013687\_contig\_number\_22213SRS016541\_contig\_number\_12137SRS022609\_contig\_number\_50241SRS023914\_contig\_number\_20660SRS045713\_contig\_number\_16102SRS049995\_contig\_number\_13351SRS054059\_contig\_number\_332SRS078176\_contig\_number\_337SRS144537\_contig\_number\_39154
species
11

2005525
family
1

195950
genus
1

species

SRS022077\_contig\_number\_contig-100\_2544.70932
712710
1
